# Turn-Directed α-β Conformational Transition of α-syn12 Peptide at Different pH Revealed by Unbiased Molecular Dynamics Simulations

**DOI:** 10.3390/ijms140610896

**Published:** 2013-05-24

**Authors:** Lei Liu, Zanxia Cao

**Affiliations:** 1 Department of Computer Science and Technology, Dezhou University, Dezhou 253023, China; E-Mail: leiliusid@gmail.com; 2 Shandong Provincial Key Laboratory of Functional Macromolecular Biophysics, Dezhou 253023, China

**Keywords:** Parkinson’s disease, α-syn12 peptide, α-β transition mechanism, different pH

## Abstract

The transition from α-helical to β-hairpin conformations of α-syn12 peptide is characterized here using long timescale, unbiased molecular dynamics (MD) simulations in explicit solvent models at physiological and acidic pH values. Four independent normal MD trajectories, each 2500 ns, are performed at 300 K using the GROMOS 43A1 force field and SPC water model. The most clustered structures at both pH values are β-hairpin but with different turns and hydrogen bonds. Turn_9-6_ and four hydrogen bonds (HB_9-6_, HB_6-9_, HB_11-4_ and HB_4-11_) are formed at physiological pH; turn_8-5_ and five hydrogen bonds (HB_8-5_, HB_5-8_, HB_10-3_, HB_3-10_ and HB_12-1_) are formed at acidic pH. A common folding mechanism is observed: the formation of the turn is always before the formation of the hydrogen bonds, which means the turn is always found to be the major determinant in initiating the transition process. Furthermore, two transition paths are observed at physiological pH. One of the transition paths tends to form the most-clustered turn and improper hydrogen bonds at the beginning, and then form the most-clustered hydrogen bonds. Another transition path tends to form the most-clustered turn, and turn_5-2_ firstly, followed by the formation of part hydrogen bonds, then turn_5-2_ is extended and more hydrogen bonds are formed. The transition path at acidic pH is as the same as the first path described at physiological pH.

## 1. Introduction

The aggregation of α-synuclein protein in the form of a β-structure is a hallmark of Parkinson’s disease [1,2]. The N-terminal region of α-synuclein plays a key role in the formation of α-synuclein assemblies [3] and binding to the coiled-coil domain of synphilin-1 [4]. Our early studies [5–7] indicated that the α-syn12 peptide adopted a β-hairpin conformation in aqueous solution, obtained in temperature replica exchange molecular dynamics (T-REMD) simulations using GROMOS 43A1 and OPLS-AA force fields. Thus, understanding how the α-syn12 peptide transforms from α-helical to β-hairpin conformation should shed light on critical initial processes in protein aggregation.

Molecular dynamics simulation is a valid method for investigating transition mechanisms of peptides. For non-amyloidogenic β-hairpin peptides, two major folding mechanisms have been proposed based on a number of experimental and computational studies [8–12]. One is a hydrophobic collapse mechanism [13], where the folding begins from the formation of native hydrophobic clusters, then proceeds with the interstrand hydrogen bond. Another is the zipper mechanism [14], the first step of which forms the turn, followed by the formation of interstrand hydrogen bond. For example, Thukrai *et al.* [15,16] investigated the folding mechanism of a 15 residue β-hairpin peptide and found that turn is always the major determinant in initiating the folding process, and supports the second folding mechanism. Yoda *et al.* [17] studied the folding mechanism of a 16 residue peptide of the C-terminal end of a GB1 domain, and the results indicated that the formation of the specific turn structure is very important. Although many reports exist on non-amyloidogenic peptides simulated at atomic resolution on the microsecond time scale, simulations for amyloidogenic peptides on the microsecond time scale are few. Daidone *et al*. and Chiang *et al*. [18–21] revealed the mechanisms of the α to β conformational transition of the Syrian hamster PrP peptide H1 and the Aβ (12–28) fragment; the simulations highlight the formation of the bent conformation. Levy *et al*. [22] observed the helix to coil conformational transition of the PrP (106–126) peptide by performing a set of 34 MD simulations. Klimov *et al.* [23] showed that the oligomerization of Aβ (16–22) requires the peptide to undergo a random coil to α helix to β transition via MD simulations. It is difficult to say whether the α-syn12 peptide shares the same folding mechanism with the H1 peptide or Aβ peptide.

Moreover, experimental studies suggest that α-synuclein aggregates at low pH faster than at neutral pH [24,25]. But the effects of different pH on transition mechanism of amyloidogenic peptides have been demonstrated in very few studies. It is therefore instructive to compare the structural character and transition paths at different pH values.

In this work, we focus on the transition mechanism of the α-syn12 peptide in explicit water at atomic resolution by long timescale unbiased molecular dynamics. Four MD trajectories, each 2500 ns long, were generated starting from α helix with different initial velocity, resulting in a combined simulation time of 10 μs. The transition mechanism was analyzed from the parameters such as the formation of β-hairpin, turn and hydrogen bonds, and the representative conformations. Particular attention has been given to answer the question whether the turn promotes the hairpin folding or the turn is driven by the hydrogen bonds. The results regarding the α-syn12 peptide transition mechanism are in favor of the turn driven process. Furthermore, two transition paths are observed at physiological pH. One of the two paths is the same as the path at acidic pH.

## 2. Results and Discussion

### 2.1. The Most Clustered Structures

Cluster is a simple and widely used method to reveal the representative conformation of α-syn12 at different pH. A total of 50,000 conformations were obtained from the trajectories during the 1–2500 ns of the two simulations at each pH and were clustered based on their mutual root-mean-square deviations of Cα positions (RMSDCα). The criterion of clustering is that the conformations are in the same cluster when RMSDCα is less than 0.1 nm among the conformations of this cluster.

**Figure 1:**
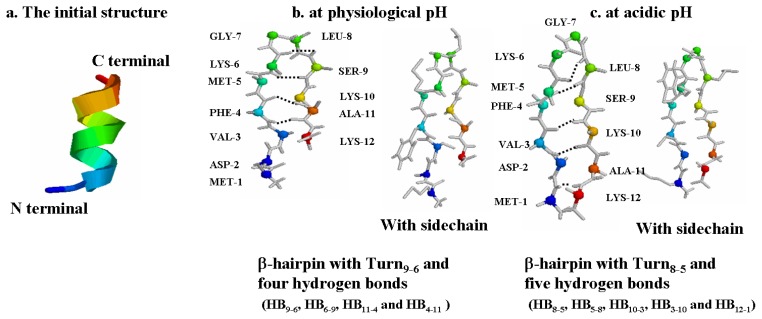
The initial structure (**a**) and the most clustered structure at physiological pH (**b**) and acidic pH (**c**). The Cα atoms are shown as spheres. The interstrand hydrogen bonds are shown with dotted lines.

The initial structure for the α-syn12 peptide (Figure 1a) is a α-helix. The most clustered structure for the α-syn12 peptide at physiological pH (Figure 1b) is a β-hairpin with Turn_9-6_ and four hydrogen bonds (HB_9-6_, HB_6-9_, HB_11-4_ and HB_4-11_); this cluster contains 89% of all the conformations. This structure was consistent with the central structure of the major clusters for the α-syn12 peptide at physiological pH from the T-REMD simulations using GROMOS 43A1 force field from our early studies [7]. However, the most clustered structure for the α-syn12 peptide at acidic pH (Figure 1c) is a β-hairpin with Turn_8-5_ and five hydrogen bonds (HB_8-5_, HB_5-8_, HB_10-3_, HB_3-10_ and HB_12-1_); this cluster contains 84% of all the conformations.

Turn_9-6_ denotes that a β-turn forming among residues 6–9. HB_4-11_ is defined as hydrogen bond between hydrogen atom of the amide NH of residue 4 and backbone carbonyl oxygen atom of residue 11. Formation of the β-turn was estimated by the program STRIDE [26]. Criteria of hydrogen bonds are that the donor-hydrogen-acceptor angle is more than 135° and the distance between the hydrogen and the acceptor atom is less than 2.5Å. The distance and angle are selected based on the analysis software GROMOS++ [27].

### 2.2. Root Mean Square Deviations of Cα Atoms (RMSDCα)

**Figure 2:**
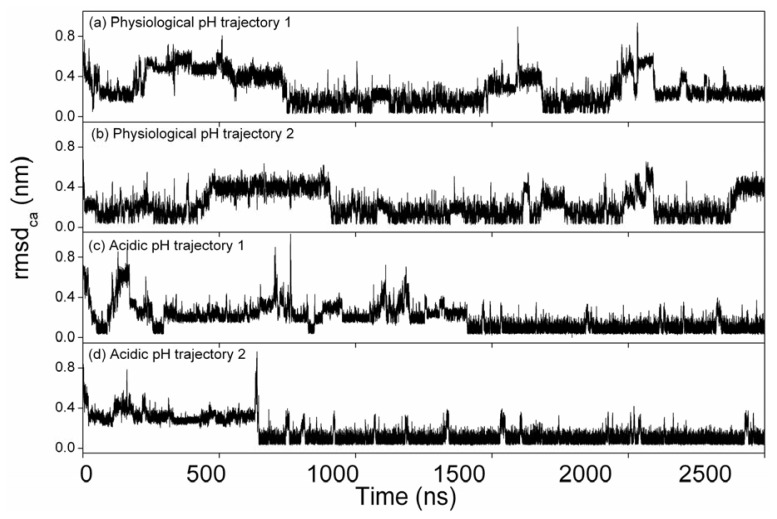
Time evolution of the positional root mean square deviations (RMSD) of alpha-carbon atoms with respect to the most clustered structures along the ten trajectories.

The atom-positional root mean square deviations (RMSD) of Cα atoms in respect to the representative structure derived from the conformation clusters for the α-syn12 peptide simulations are shown in Figure 2 as a function of the simulation time. The peptide folds into the β-hairpin structure in all simulations.

### 2.3. Residue-Specific Secondary Structure Propensity

**Figure 3:**
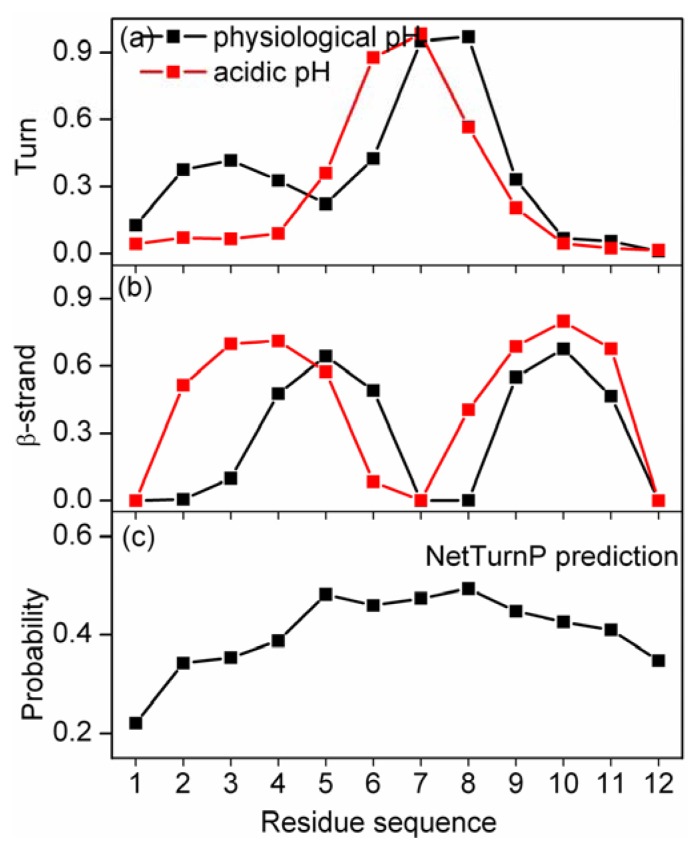
Turn (**a**) and β-strand (**b**) occurrence probabilities for α-syn12 peptide at physiological pH (black) and acidic pH (red). The NetTurnP prediction probability (**c**).

The residue-specific secondary structure propensities of this peptide at different pH were calculated with the program STRIDE. The results are shown in Figure 3a,b. At physiological pH, two highly populated turn structures were observed, centered at residues 2–5 and 6–9; one highly populated β-strand structure centered at residues 4–6 and 9–11 was observed and four hydrogen bonds were formed. However, at acidic pH, only one populated turn structure centered at residues 5–8 was observed; one highly populated β-strand structure centered at residues 2–5 and 8–11 was observed and five hydrogen bonds were formed. Residues 2–4 display more turn structure at physiological pH than acidic pH.

Residues such as Asn, Asp, Gly, Pro and Ser tend to form turns. In this sequence, the probabilities of each residue to form turns can be predicted with the software NetTurnP [28] and the results are shown in Figure 3c. At physiological pH, Asp2 is negatively charged, which experiences electrostatic attraction from Lys6. However, at acidic pH, Asp2 is neutral. This makes it difficult to form Turn_5-2_ at acidic pH.

The above analyses are based on the program STRIDE. Another widely used secondary structure definition program is DSSP [29]. The main difference between STRIDE and DSSP is that while STRIDE considers both hydrogen bonding patterns and backbone geometry, DSSP only considers the hydrogen bonding patterns. The residue-specific secondary structure propensities of this peptide at different pH were also calculated with the program DSSP and shown in Figure S1. The two programs both indicated that the α-syn12 peptide adopts different β-hairpin structures at different pH. However, some of the turn structures determined by STRIDE are considered as bend structures with DSSP.

### 2.4. The Relationship between Turns and Hydrogen Bonds

**Figure 4:**
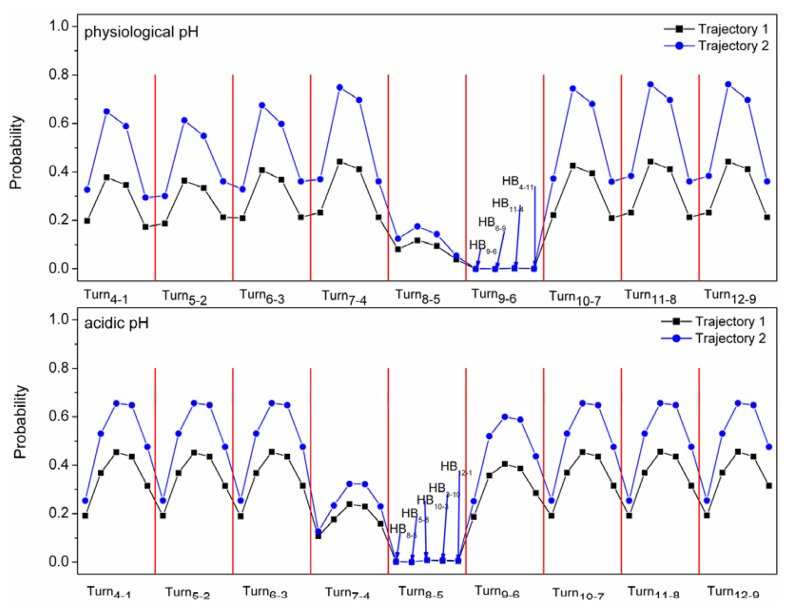
The probabilities of forming different hydrogen bonds when the Turn (*X*-axis) does not form.

The β-hairpin configuration can be produced in the simulations for the α-syn12 peptide using the GROMOS 43A1 force field. Hydrogen bond and β-turn are two important factors involved in the folding for β-hairpin structure. The probabilities of forming turn and hydrogen bonds at different pH are shown in Table 1. Although the simulations at different pH produce different turns and hydrogen bonds, the probabilities of conformation forming four hydrogen bonds are lower than the probabilities of conformation forming turns. In order to analyze the interplay between the formation of the turns and hydrogen bonds, the probabilities of conformation forming hydrogen bonds when the turns do not form were analyzed in detail and shown in Figure 4. For simulations at physiological pH, as described in Figure 4, it is shown that when turn_9-6_ does not form, the probability of conformation forming hydrogen bonds is zero for all the simulations. The other turns do not indicate this probability. The results showed that turn_9-6_ always forms before the formation of hydrogen bonds. The simulations at acidic pH also suggested that turn_8-5_ always forms before the formation of hydrogen bonds. This occurred in all four trajectories and indicates that the formation of the turn drives the folding process

### 2.5. Free Energy Surface and the Transition Path

**Figure 5:**
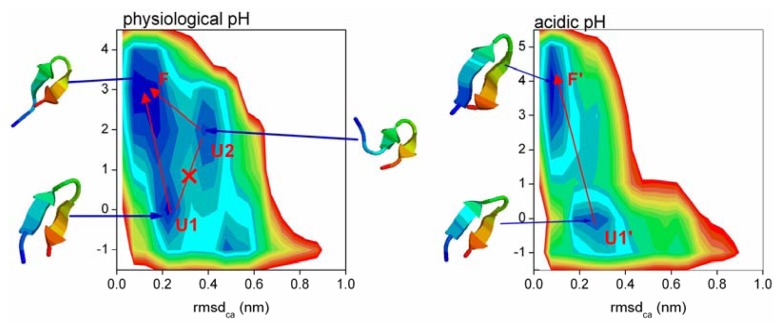
Free energy surfaces along the positional root mean square deviations of C_α_ atoms (RMSDC_α_) from the most clustered conformation and the turn formation and number of hydrogen bonds. The *Y*-axis represent the existence of the following at physiological pH: (−1) Turn_9-6_ does not form, (0) Turn_9-6_ forms but the four hydrogen bonds do not form, (1–4) Turn_9-6_ forms and the number of the four hydrogen bonds is 1–4. The *Y*-axis represents the existence of the following at acidic pH: (−1) Turn_8-5_ does not form, (0) Turn_8-5_ forms but the five hydrogen bonds do not form, (1–5) Turn_8-5_ forms and the number of the five hydrogen bonds is 1–5. Neighboring contour lines are separated by 1 kJ/mol. The representative structures are shown on the top.

The above analysis suggested that the α-β transition is initiated at the turn and is highly consistent for both pH levels. How does the peptide find the most clustered conformation during its search? To address this question, the free energy surfaces (FESs) were constructed based on two reaction coordinates (see Figure 5), the positional root mean square deviations of Cα atoms (RMSDCα) from the most clustered structure and the turn formation and number of hydrogen bonds. At physiological pH, the most clustered conformation is a β-hairpin with Turn_9-6_ and four hydrogen bonds (HB_9-6_, HB_6-9_, HB_11-4_ and HB_4-11_). We set the *Y*-axis to represent the existence of the following: (−1) Turn_9-6_ does not form, (0) Turn_9-6_ forms but the four hydrogen bonds do not form, (1–4) Turn_9-6_ forms and the numbers of the four hydrogen bonds are 1–4. Similarly at acidic pH, we set the *Y*-axis to represent the existence of the following: (−1) Turn_8-5_ does not form, (0) Turn_8-5_ forms but the five hydrogen bonds do not form, (1–5) Turn_8-5_ forms and the numbers of the five hydrogen bonds are 1–5.

At physiological pH, three local minima were obtained: U1 located at (0.23 nm, 0); U2 located at (0.38 nm, 2); and F located at (0.08 nm, 3), the relative depths of the three minima were 0.3, 1.7 and 0 kJ·mol^−1^. However, only two minima were observed at acidic pH: U1′ centered near (0.27 nm, 0); F′ centered near (0.08 nm, 4), the relative depths of the two minima were 1.8 and 0 kJ·mol^−1^.

To analyze the structural features of each minimum, we performed a RMSD based clustering analysis. The representative structures of each minimum are presented in Figure 5. At physiological pH, the representative structure of U1 is a β-hairpin with two turns (Turn_9-6_ and Turn_8-5_) and two non-most-clustered hydrogen bonds (HB_10-4_ and HB_4-10_); the representative structure of U2 is a β-hairpin with two turns (Turn_9-6_ and Turn_5-2_) and two hydrogen bonds (HB_9-6_ and HB_6-9_); the representative structure of F is a β-hairpin with Turn_9-6_ and three hydrogen bonds (HB_6-9_, HB_11-4_ and HB_4-11_). At acidic pH, the representative structure of U1′ is a β-hairpin with two turns (Turn_9-6_ and Turn_8-5_) and two non-most-clustered hydrogen bonds (HB_10-4_ and HB_4-10_); the representative structure of F′ is a β-hairpin with Turn_8-5_ and four hydrogen bonds (HB_5-8_, HB_10-3_, HB_3-10_ and HB_12-1_).

**Figure 6:**
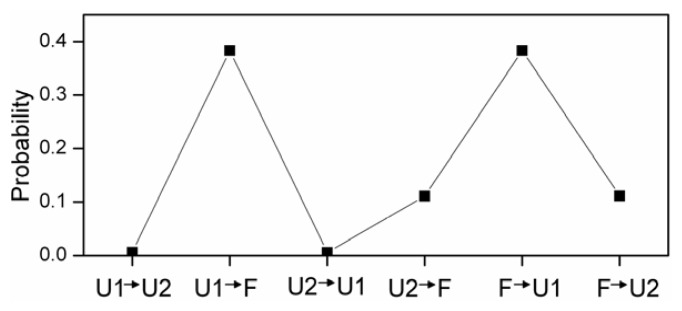
The probabilities of six different transition paths.

**Figure 7:**
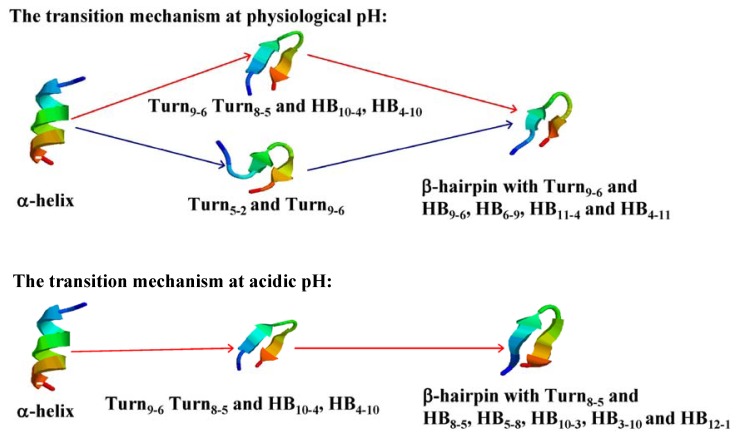
The schematic representation of the transition mechanisms at different pH.

At physiological pH, four possible transition paths: from U1 to F; from U2 to F; from U1 to U2 and last to F; from U2 to U1 and last to F. In order to examine what transition paths occurred in these simulations, the probabilities of six different ways were calculated (see Figure 6). The transition from U1 to U2 or U2 to U1 does not take place. There have two transition paths of α-syn12 peptide at physiological pH: one is from U1 to F, the most-clustered turn and improper hydrogen bonds are formed, followed by the formation of most-clustered hydrogen bonds; another is from U2 to F, the most-clustered turn and turn_5-2_ are formed, followed by the formation of hydrogen bonds, finally, turn_5-2_ are extended and more hydrogen bonds are formed. The schematic representation of the transition mechanisms at different pH is shown in Figure 7.

## 3. Methods

The initial α-helix structure (Figure 1a) for the α-syn12 peptide with a sequence of MDVFMKGLSKAK (residues 1–12 of the human α-synuclein protein) was selected from the NMR determined micelle bound structure at neutral pH (PDB ID: 1XQ8). MD simulation in the isothermal-isobaric (NPT) ensemble was performed using the GROMACS software package [30]. The GROMOS 43A1 [31] force field with the SPC [32] water model was considered herein. The peptide was solvated in a rectangular box with the minimum solute-box boundary distance being set to 1.0 nm. The long-range electrostatic interaction was treated with the particle-mesh Ewald method with a grid spacing of 0.12 nm and a fourth order interpolation [33,34]. Protonation states of ionizable groups were chosen for physiological pH and acidic pH. At physiological pH, three lysine residues side chain protonated and aspartate residue side chain deprotonated, two negative counterions (Cl-) were added to produce a neutral simulation system. The simulation system contained 1710 water molecules. At acidic pH, three lysine residues side chain protonated and aspartate residue side chain protonated, and three negative counterions (Cl-) were added to produce a neutral simulation system. The simulation system contained 1708 water molecules.

At each pH, two independent simulations with different initial velocities were performed. Each simulation trajectory was run for 2500 ns at the temperature *T* = 300 K and the pressure *P* = 1 bar. The temperature of the system was kept constant by using velocity rescaling with a stochastic term [35]. The pressure of the system was kept constant by using a weak coupling algorithm [36]. The simulation was made using a temperature coupling time of 0.1 ps and pressure coupling time of 0.5 ps, and an isothermal compressibility of 4.575 × 10^−4^ (kJ·mol^−1^·nm^−3^)^−4^. The time step for the MD integrator was set to 2 fs and LINCS [37] was applied to constrain all bond lengths.

## 4. Conclusions

Parkinson’s disease is characterized by the deposition of aggregated fibrillar α-synuclein in Lewy bodies within the brain. Experimental studies [24,25,38] suggested that low pH values stimulate the aggregation of α-synuclein. Additionally, the N-terminal region of α-synuclein plays a key role in the formation of α-synuclein assemblies. In order to understand the pathological mechanism of Parkinson’s disease at the molecular level and explore the possible factor which can regulate α-synuclein assembly, it is necessary to reveal the structural character and the conformational transitions of α-syn12 peptide under different conditions.

A series of our early studies [5–7] and this study indicated that the α-syn12 peptide adopts different most clustered structure at different pH levels. At physiological pH, the most clustered structure is a β-hairpin with Turn_9-6_ and four hydrogen bonds (HB_9-6_, HB_6-9_, HB_11-4_ and HB_4-11_), with two hydrophobic residues (Phe4 and Ala11) involved in the formation of hydrogen bonds. At alkaline pH, the most clustered structure is a part β-hairpin with Turn_9-6_ and two hydrogen bonds (HB_6-10_ and HB_10-6_), with no hydrophobic residues involve in the formation of hydrogen bonds. At acidic pH, the most clustered structure is a β-hairpin with Turn_8-5_ and five hydrogen bonds (HB_8-5_, HB_5-8_, HB_10-3_, HB_3-10_ and HB_12-1_), and four hydrophobic residues (Met1, Val3, Met5 and Leu8) involved in the formation of hydrogen bonds. These results might explain why α-synuclein aggregates at acidic pH faster than at neutral pH. The acidic pH increases the number of the inter-peptide hydrogen bonds. More experiments or molecular simulations on dimer or oligomer are needed to explore the factors that influence the aggregation.

Another aim of the present study was to compare the transition mechanism of α-syn12 peptide at different pH values. A common folding mechanism was observed that indicates that the formation of the turn drives the folding process. A transition path at both physiological pH and acidic pH forms the most-clustered turn and improper hydrogen bonds firstly. Another transition path, at physiological pH tends to form the most-clustered turn and turn_5-2_, firstly.

## Supplementary Information



## Figures and Tables

**Table 1:** The respective probabilities of α-syn12 peptide forming turns and four hydrogen bonds.

	Turn_9-6_	HB_9-6_	HB_6-9_	HB_11-4_	HB_4-11_	
Trajectory 1 at physiological pH	0.81	0.23	0.44	0.41	0.21	
Trajectory 2 at physiological pH	0.98	0.38	0.76	0.70	0.36	

	**Turn_8-5_**	**HB_8-5_**	**HB_5-8_**	**HB_10-3_**	**HB_3-10_**	**HB_12-1_**

Trajectory 1 at physiological pH	0.86	0.19	0.37	0.46	0.44	0.31
Trajectory 2 at physiological pH	0.66	0.18	0.36	0.45	0.44	0.48
