# Supplementary material for: Turn-Directed α-β Conformational Transition of α-syn12 Peptide at Different pH Revealed by Unbiased Molecular Dynamics Simulations
Source: Int J Mol Sci. 2013 May 24;14(6):10896–907. doi: 10.3390/ijms140610896 (PMC3709708; doi:10.3390/ijms140610896)
Supplement: Supplementary file 1 [file ijms-14-10896-s001.pdf]

Supplementary Information

Figure S1. Turn (**a**), β-strand (**b**) and bend (**c**) occurrence probabilities for α-syn12 peptide at physiological pH (black) and acidic pH (red) with program DSSP.

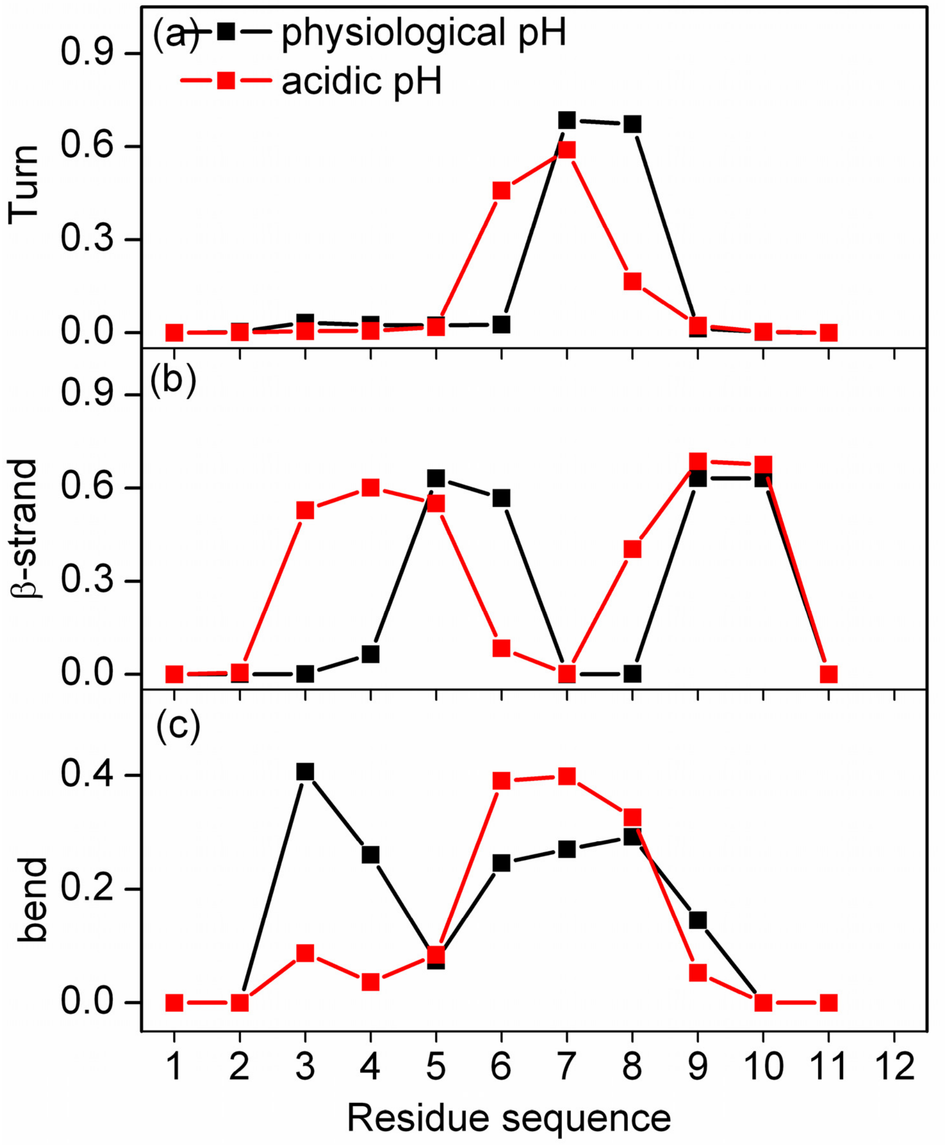

© 2013 by the authors; licensee MDPI, Basel, Switzerland. This article is an open access article distributed under the terms and conditions of the Creative Commons Attribution license (http://creativecommons.org/licenses/by/3.0/).
